# 3D Morphology Analysis of TMJ Articular Eminence in Magnetic Resonance Imaging

**DOI:** 10.1155/2017/5130241

**Published:** 2017-06-21

**Authors:** Izabella Nascimento Falcão, Maria Beatriz Carrazzone Cal Alonso, Lucas Hian da Silva, Sérgio Lúcio Pereira de Castro Lopes, Lívia Pichi Comar, André Luiz Ferreira Costa

**Affiliations:** ^1^Department of Orthodontics and Radiology, University of São Paulo City (UNICID), São Paulo, SP, Brazil; ^2^Department of Diagnosis and Surgery, São José dos Campos Dental School, São Paulo State University (UNESP), São José dos Campos, SP, Brazil

## Abstract

**Purpose:**

The objective of this study was to evaluate the computational reconstruction of the articular eminence of the temporomandibular joint (TMJ) based on magnetic resonance imaging (MRI) and semiautomatic volumetric segmentation techniques for morphological classification of the TMJ structure.

**Materials and Methods:**

A total of 36 MRI scans of TMJ individuals were selected and formatted by using the ITK-SNAP software, consisting of MRI segmentation and generation of 3D models. The TMJ articular eminences were also classified according to the morphology analysis of the articular eminence in 3D reconstructions. Two independent trained and calibrated investigators performed the image analysis, which was repeated after thirty days.

**Results:**

There was no association between sex and eminence shape (*p* = 0.456). Fisher's test revealed no statistically significant association between disc classification and eminence shape on both sides (*p* = 0.629). Chi-square test showed a significant statistically association between disc classification and disc displacement (*p* = 0.000). Intra- and interrater correlation coefficients showed excellent reproducibility values.

**Conclusions:**

Anatomical variability of the sample investigated was found, with predominantly round shape and presence of correlation between this shape and normal disc position. The correlation of flattened and convex shapes with disc position reduction indicated that type of disc derangement is more prevalent.

## 1. Introduction

The temporomandibular joint (TMJ) has a disc located between two bones composed of mandibular fossa and articular eminences of the temporal bone and mandibular condyle [1]. The complexity of TMJ, with anatomic and biomechanical involvement, increases its susceptibility to pathological changes [2].

The articular eminence has an important role in the biomechanics of the TMJ [3–5], with some authors suggesting that its morphology may be a factor in the etiology of temporomandibular disorders (TMD). Also, the eminence shape has an effect on determining whether a displaced disc is reducible; it may be reducible when it returns spontaneously to the glenoid cavity, or nonreducible when the disc remains dislocated [3, 6–11].

The morphological evaluation of TMJ bony structures is object of interest in the improvement of the image quality in CT and especially MRI for a reliable diagnosis of TMD [12]. The TMD diagnosis may pose a challenge to the dentist/radiologist, especially when considering the size and degree of involvement of the joint [13]. Moreover, there are overlapping findings with other joint changes, especially degenerative processes. Magnetic resonance imaging (MRI) has been the preferred method to aid in the diagnosis of various TMJ pathologies [14–17]. This is due in part to the wealth of details provided by MRI compared to other imaging methods, including the fact that this technique is not limited to produce images of cross-sections and there is nonionizing radiation [18], as is the case with other modalities such as CT [12]. However, the spatial resolution of MRI is not sufficient for accurate images of the entire TMJ due to contrast variation, resulting in inconspicuous or invisible images of the anatomy and thus leading to misdiagnosis [19], mainly in the bone tissue [20, 21].

Three-dimensional (3D) reconstruction model based on MRI can play an important role in the TMD diagnosis [19, 21, 22] by revealing the morphological features of TMJ with simplicity, speed, accuracy, and limited user interaction [23], thus being a powerful tool for characterizing different patterns of TMJ pathologies [24].

The purpose of this article is to demonstrate the use of 3D model to classify the morphology of the articular eminence by correlating signs and symptoms of TMD to articular disc displacement on MRI images.

## 2. Material and Methods

### 2.1. Image Data

This study has been conducted in accordance with universally accepted rules and precepts for research involving human subjects, being approved by the local research ethics committee of the University of São Paulo City.

From an initial sample of 60 individuals, we have selected a set of images from 36 individuals (28 women, with mean age of 37.79 ± 15.68, and 8 men, with mean age of 37.63 ± 1.42) attending the Clinic Hospital of the University of Campinas (UNICAMP). Images showing the region of interest not clearly enough were discarded. The images were acquired on a commercially available 2 Tesla scanner (Elcint Prestige, Haifa, Israel) with bilateral surface coils of 40 mm in diameter dedicated to TMJ imaging, according to the following parameters: T1-weighted sagittal images (TR = 650 msec, TE = 22 msec, matrix = 316 × 240.160, flip angle, thickness of 1.5 mm, field of view = 10 cm × 10 cm, NEX 1, voxel size 0.29 mm × 0.29 mm × 0.3 mm voxel) were acquired at spin-echo sequence with mouth closed.

### 2.2. MRI Segmentation and Generation of 3D Models

**Figure 1:**
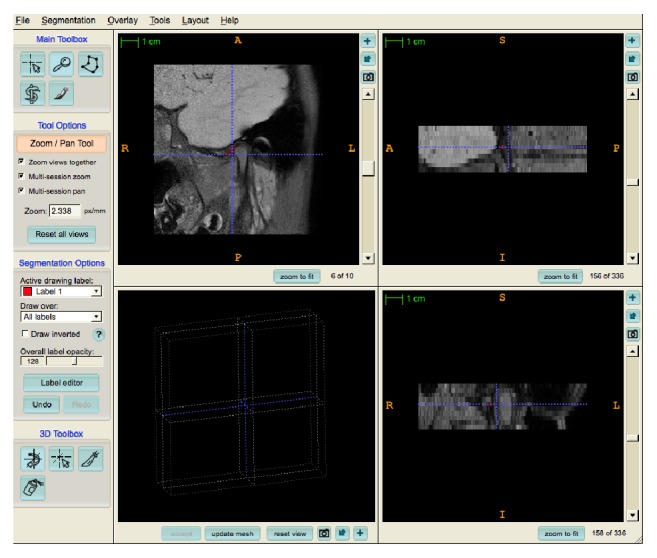
Software interface showing delimitation of the articular eminence in the sagittal plane on MRI.

The MRIcro software (http://www.mricro.com) was used to convert the original Digital Imaging and Communication in Medicine (DICOM) format into ANALYZE format. After format conversion, the ITK-SNAP software [25] (http://www.itksnap.org/download/snap) was used for segmentation. This software can provide semiautomatic segmentation using level set methods, as well as manual delineation and image navigation [26, 27]. A 3D model of the target structure was generated after segmentation, thus allowing us to see the object from any view point by zooming, rotating, and panning [27] (Figure 1). The 3D-graphical model of the right and left articular eminences was generated by the software, in which the cortical bone was delineated with semiautomatic discrimination of the structures to allow for manual editing and slice-by-slice review in all three planes.

Two independent trained researchers used semiautomatic segmentation to delineate the articular eminence edge and to define image voxels and boundary [25]. The segmentation resulted in a 3D model of the bone.

### 2.3. Morphological Assessment of the Joint

**Figure 2:**
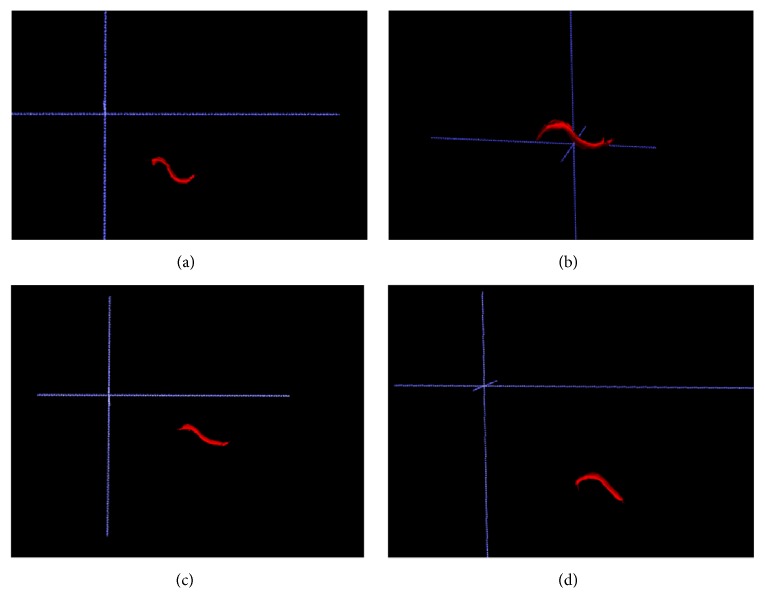
3D reconstruction of the articular eminence characterizing it as (a) box, (b) sigmoid, (c) flattened, and (d) deformed.

Each evaluator performed the morphological analysis of the articular eminence in the 3D reconstructions (Figure 2), classifying them into four types according to criteria by Kurita et al. [10]:

- Box
- Sigmoid
- Flattened
- Deformed

In assessing 3D models, the evaluator could visualize the articular eminence from any view point. The evaluation was repeated by each examiner within an interval of 30 days.

### 2.4. Statistical Analysis

All the information obtained was analysed by using the SAS 9.4 and Minitab 16 statistical software, with concordance analysis being performed in two moments for eminence classification. First, the agreement between both evaluations of the eminence classifications was analysed on the left side, and then the same analysis was performed for evacuations on the right side. Repetition agreement and inter- and intrarater reliability analysis were performed by using Fleiss' kappa, Cohen's kappa, and Kendall's coefficients. All analyses were conducted at confidence level of 95%.

## 3. Results

Table 1 shows that no significant statistical difference was observed between age groups (*p* = 0.978), regardless of sex. Our results showed that age was not a determining factor for TMJ disc shape. For analysis of the eminence classification on the left side, the results showed Fleiss' kappa value = 0.79877, Cohen's kappa value = 0.79955, and Kendall's coefficient value = 0.793050. For evaluations on the right side, the results showed Fleiss' kappa value = 0.88060, Cohen's kappa value = 0.88119, and Kendall's coefficient value = 0.839011.

**Figure 3:**
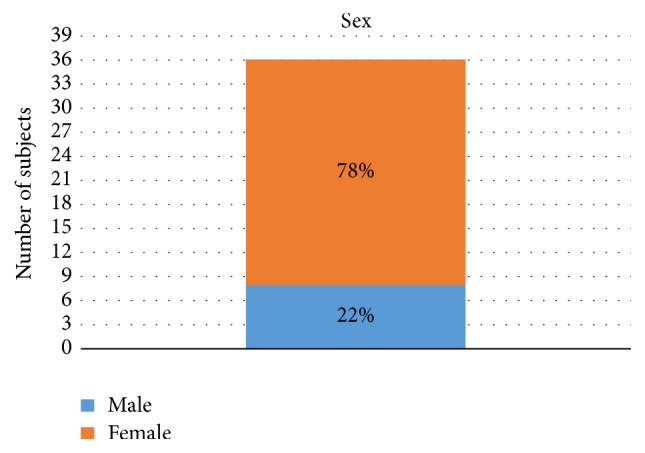
Distribution of the sample according to sex.

**Figure 4:**
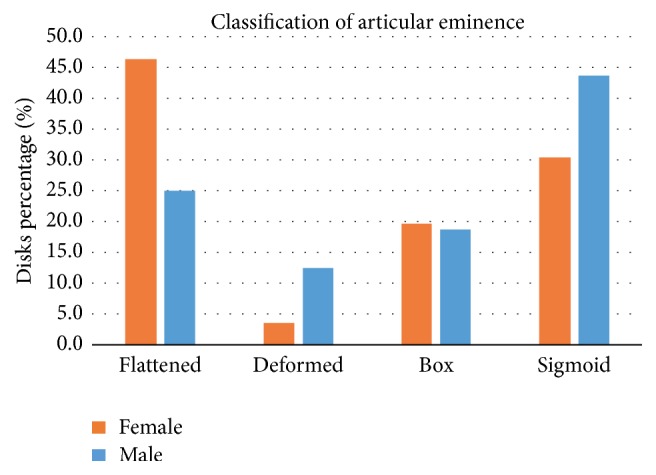
Distribution of the sample according to sex and articular eminence morphology.

Figure 3 shows that there was a significant difference between sexes, since women corresponded to 78% of the sample.  Figure 4 shows the results of Fisher's exact test performed with Freeman-Halton extension. Our intention was to verify whether sex has any association with the articular eminence shape. The shapes observed on the left and right sides of men and women were added. There was no association between sex and eminence shape (*p* = 0.456).

**Figure 5:**
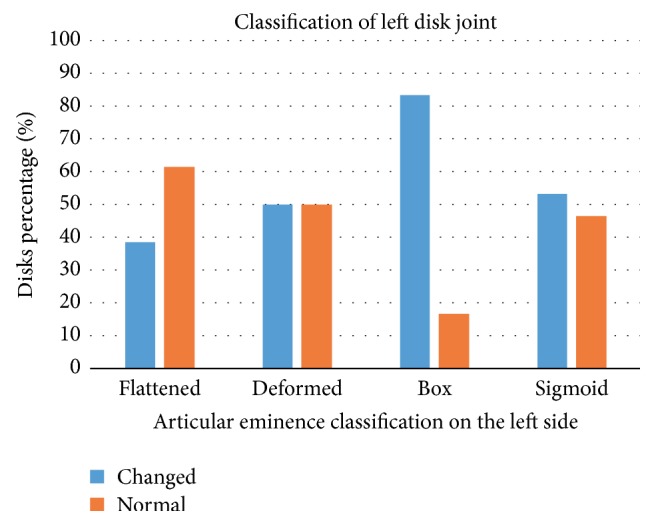
Association between the morphology of the left articular eminence with the disk classification on the left side.

**Figure 6:**
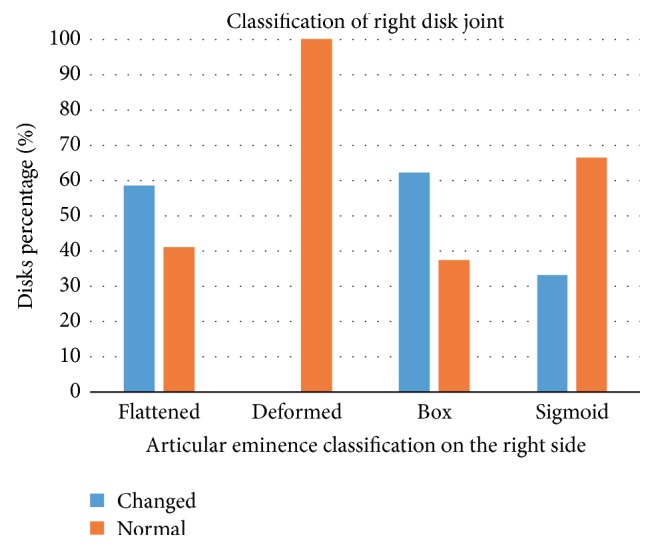
Association between the morphology of the articular eminence and disk classification on the right side.

Figures 5 and 6 show the results of Fisher's exact test performed with Freeman-Halton extension. There was no statistically significant association between disc classification and eminence shape on the left side (*p* = 0.629) as well as on the right side (*p* = 0.629).

**Figure 7:**
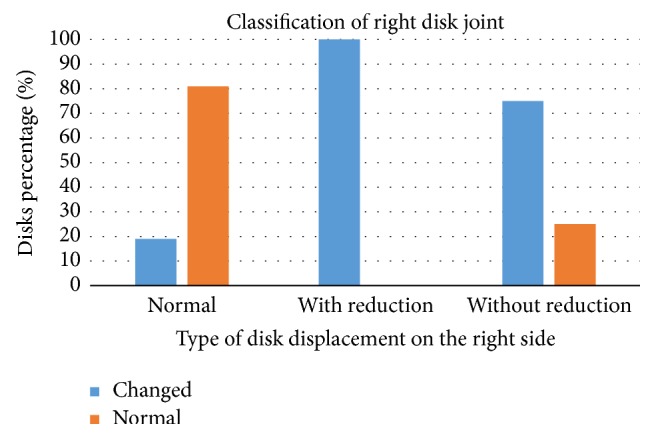
Association between disk classification and disc classification on the right side.

**Figure 8:**
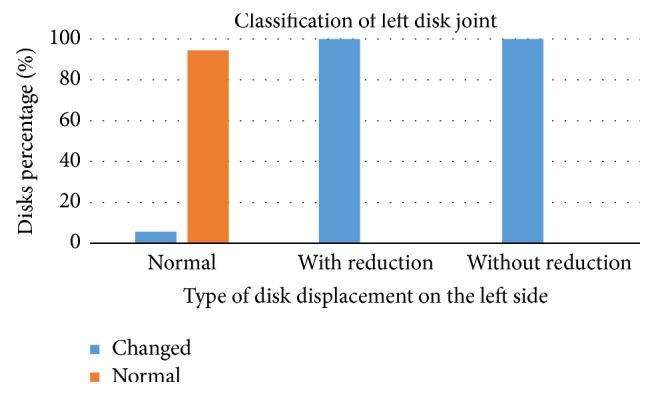
Association between disc classification and classification of disc displacement on the left side.

Figures 7 and 8 show the results of Chi-square test (*χ*^2^) in which a significant association was found between disc classification and disc displacement classification on the right side (*p* = 0.000) as well as on the left side (*p* = 0.000).

## 4. Discussion

It is important to correctly identify bone abnormalities in the TMJ because there are anatomical variations depending on both patient and influences of clinical factors. MRI is a widely used tool for diagnosis of TMDs, but because of the lack of contrast between different tissues, it may not be precise enough for analysis of structures [19], especially in bone tissues [20]. It has been reported that the three-dimensional (3D) reconstruction model based on MRI can play an important role in TMD diagnosis [19, 21, 22] by revealing morphological features of TMJ [23], thus being a powerful tool for characterizing different patterns of TMJ pathologies [24]. Therefore, the present study aimed to demonstrate the use of 3D model to classify the morphology of the articular eminence by correlating signs and symptoms of TMD to articular disc displacement on MRI images and clarify whether 3D reconstruction can be used safely and reliably.

Hirata et al. (2007) showed in their study that some alterations in the morphology of both articular eminence and disc may influence the occurrence of nonreducing disc displacement on the affected side. Changes in occlusion (i.e., missing teeth, orthognathic surgery, and orthodontics), parafunctional habits, and positioning changes between articular disc and mandibular condyle can contribute to the remodeling of the joint structures in the TMJ [1, 6]. The authors defined that the main mechanical factors causing changes in the TMJ structure are occlusal therapy, parafunction, macrotrauma, and unstable occlusion. For the authors, these factors can occur alone or interconnectedly. When two or more than 11 factors are present, it is more likely that morphological changes occur, or if the patient has limited bone adaptation capacity, condylar changes will be more pronounced. Based on our results, it can be observed that of a total of 36 patients studied, 71.4% had articular eminence in the form of box, 50% had flattened articular eminence, 43.9% had sigmoid articular eminence, and 25% had deformed articular eminences, all presenting alterations in the shape of the articular disc. Dental occlusion plays an important role as a predisposing factor, since it changes the masticatory system enough to increase the risk of developing TMJ dysfunction [9]. Parafunctional habits and dental malocclusion induce the production of microtraumas in TMJ by causing degenerative lesions in the condyle and articular disc, which in turn tend to be thickened to compensate for the resulting damage [7].

Our results have shown that no association was found between gender and eminence shape based on the eminence classification (*p* value = 0.456). According to Figure 4, it was noticed that the shape variation happens in a similar way in both genders, although alterations occurred more in women than in men (as expected), a finding corroborated by Hirata (2007) and Kurita (2000).

The results of intrarater variation with Cohen's kappa statistics suggest that reproducibility was very good, having a high standard of agreement.

We agree that 3D models are valuable for improving the visualization of anatomical structures [22, 25, 26]. The experimental results revealed that 3D reconstruction approach can generate an accurate 3D model and thus can assist the dental radiologist in the diagnosis of articular eminence. Although dental radiologists are trained to interpret MRI scans, they often find difficulty to analyse them [24].

## 5. Conclusions

According to the methodology used, it can be concluded that there is a relationship between the presence of changes in the articular discs and shape of their articular eminence. Thus, a higher incidence of deformations in articular eminences was classified as being of box shape. We emphasize the relevance of our findings and the importance of carrying out complementary studies into this field so that our findings can be replicated in larger samples.

## Figures and Tables

**Table 1:** Analysis of the comparison between sexes.

Sex	*n*	Age (years)	Groups^*∗*^	*p* value
Female	28	37.79 ± 15.68	A	0.978
Male	8	37.63 ± 13.42	A	

^*∗*^Equal letters represent statistical similarity at the significance level of 5% by Student's *t*-test.
